# Rapamycin and FTY720 Alleviate Atherosclerosis by Cross Talk of Macrophage Polarization and Autophagy

**DOI:** 10.1155/2018/1010248

**Published:** 2018-12-06

**Authors:** Rui-zhen Sun, Ying Fan, Xiao Liang, Tian-tian Gong, Qi Wang, Hui Liu, Zhi-yan Shan, Lei Lei

**Affiliations:** ^1^Department of Histology and Embryology, Harbin Medical University, 150081, Harbin, China; ^2^Department of Cardiology, First Affiliated Hospital of Harbin Medical University, 150081, Harbin, China

## Abstract

Foam cell formation and macrophage polarization are involved in the pathologic development of atherosclerosis, one of the most important human diseases affecting large and medium artery walls. This study was designed to assess the effects of rapamycin and FTY720 (fingolimod) on macrophages and foam cells. Mouse peritoneal macrophages were collected and treated with rapamycin and FTY720 to study autophagy, polarization, and lipid accumulation. Next, foam cells were formed by oxidizing low-density lipoprotein to observe changes in lipid accumulation, autophagy, and polarization in rapamycin-treated or FTY720-treated foam cells. Lastly, foam cells that had been treated with rapamycin and FTY720 were evaluated for sphingosine 1-phosphate receptor (S1prs) expression. Autophagy microtubule-associated protein 1 light chain 3- (LC3-) II was increased, and classically activated macrophage phenotype markers interleukin- (IL-) 6, cyclooxygenase-2 (COX2), and inducible nitric oxide synthase (iNOS) were increased, whereas alternatively activated macrophage phenotype markers transforming growth factor- (TGF-) *β*, arginase 1 (Arg1), and mannose receptor C-type 1 (Mrc1) were decreased by rapamycin in peritoneal macrophages. LC3-II was also obviously enhanced, though polarization markers were unchanged in rapamycin-treated foam cells. Moreover, lipid accumulation was inhibited in rapamycin-treated macrophage cells but was unchanged in rapamycin-treated foam cells. For FTY720, LC3-II did not change, whereas TGF-*β*, Arg1 and Mrc1 were augmented, and IL-6 was suppressed in macrophages. However, LC3-II was increased, and TGF-*β*, ARG1 and MRC1 were strikingly augmented, whereas IL-6, COX2 and iNOS could be suppressed in foam cells. Furthermore, lipid accumulation was alleviated in FTY720-treated foam cells. Additionally, S1pr1 was markedly decreased in foam cells (*P* < .05); S1pr2, S1pr3, S1pr4 and S1pr5 were unchanged in rapamycin-treated foam cells. In FTY720-treated foam cells, S1pr3 and S1pr4 were decreased, and S1pr1, S1pr2 and S1pr5 were unchanged. Therefore, we deduced that rapamycin stimulated classically activated macrophages and supressed early atherosclerosis. Rapamycin may also stabilize artery plaques by preventing apoptosis and S1PR1 in advanced atherosclerosis. FTY720 allowed transformation of foam cells into alternatively activated macrophages through the autophagy pathway to alleviate advanced atherosclerosis.

## 1. Introduction

Atherosclerosis, one of the most harmful human diseases of large and medium artery walls, leads to acute myocardial infarction and sudden death [1]. It has been demonstrated that atherosclerosis involves lipid accumulation and inflammatory infiltration [1], and that macrophages play a crucial role in pathogenesis. During the initial phase of atherosclerosis development, circulating monocytes migrate into the arterial wall via dysfunctional endothelial cells and then differentiate into macrophages [2–4]. Next, macrophages engulf oxidized low-density lipoprotein (ox-LDL) to digest and transport lipids out of the vascular wall [5]. When overloaded with lipid droplets, macrophages will transform into foam cells that initiate plaque formation inside the blood vessels [6]. This inflammatory process appears to be a hallmark of atherosclerosis [7–9]. Thus, decreasing macrophage foam cell formation would be an attractive strategy for reversing atherosclerosis.

Macrophage phenotype emerges in response to the microenvironment in a process referred to as macrophage activation or polarization [10]. Macrophages are either classically activated (M1) or alternatively activated (M2). M1 macrophages are activated by treatment with interferon-*γ* or lipopolysaccharide, whereas M2 macrophages are activated by treatment with Th2 cytokines interleukin- (IL-) 4 or IL-13; the M2 phenotype switch can be enhanced by IL-10. Early in the innate immune response, M1 macrophages produce reactive oxygen species and proinflammatory cytokines and chemokines to drive inflammation; thus, they are referred to as “killer” macrophages. During the resolution phase of inflammation, M2 macrophages scavenge debris and assist in angiogenesis and wound healing; thus, they are referred to as “healer” macrophages [11]. During atherosclerosis development, there is differential polarization of macrophages that results in differences in the number and distribution of polarization macrophages within the plaque. M1 and M2 macrophages link to produce atherosclerotic plaques, and the M2 macrophages can resist foam cell transformation [2]. Thus, selective removal of macrophages or altering polarization status within the plaque may have a role in alleviating atherosclerosis.

2-Amino-2-[2-(4-octylphenyl)ethyl]propane-1,3-diol hydrochloride (FTY720), also known as fingolimod, is an immune-modulating drug used to treat multiple sclerosis and multiple organ transplantation. It is both a synthetic sphingosine 1-phosphate (S1P) analogue and an S1P receptor modulator [12]. The drug may serve as a functional antagonist or agonist, depending on the S1P receptor subtype and target cell or tissue. S1P induces M2 phenotype polarization via IL-4 to protect against atherosclerosis development [13]. Some studies have shown that FTY720 reduces atherosclerosis by suppressing monocyte/macrophage migration to atherosclerotic lesions [14]. Short-term, low-dose oral FTY720 has shown great benefit in inhibiting early development of atherosclerosis via induction of regulatory T-cells and inhibition of effector T-cell response in apolipoprotein E-deficient mice fed a high-cholesterol diet [15]. Moreover, FTY720 treatment of low-density lipoprotein receptor- (LDLR-) deficient mice fed a cholesterol-rich diet activates M2 phenotype marker IL-4 in peritoneal macrophages to reduce atherosclerotic lesion formation in a dose-dependent manner. Concentrations of proinflammatory cytokines such as tumor necrosis factor-*α*, IL-6, and IL-12 are also reduced [12]. However, FTY720 failed to affect atherosclerosis in moderately hypercholesterolemic LDLR^−^/^−^ mice [16]. Thus, some important questions remain regarding how FTY720 affects macrophage function and whether FTY720 plays a role in alleviating atherosclerosis through interaction with foam cells.

Autophagy is an evolutionarily conserved, physiologic, self-protective process. Autophagy is classically considered a pathway that contributes to cellular homeostasis and adaptation to stress [17]. Dysfunctional autophagy is associated with some human diseases. A limited number of clinical studies have shown that autophagy is impaired in the advanced stages of atherosclerosis and that its deficiency induces lethal accumulation of cholesterol crystals and promotes atherosclerosis [18–20]. Stents eluting the mTOR inhibitor, everolimus, selectively clear macrophages by autophagy in rabbit model atherosclerotic plaques to promote stability without affecting smooth muscle cell viability [21]. Several examinations have also shown that mice with Atg5 (an essential autophagy gene) macrophage-specific deletion develop plaques characterized by increased apoptosis, oxidative stress, and plaque necrosis [22]. These results suggest that macrophage autophagy plays an essential but complicated role in the pathogenesis of atherosclerosis. Moreover, in vivo experiments have shown that rapamycin, an mTOR inhibitor, reduces macrophage death rate and delays plaque progression through autophagy upregulation [23]. In vitro experiments show that rapamycin not only reduces intracellular lipid droplet accumulation, but also inhibits cell apoptosis by clearing dysfunctional mitochondria and lowering intracellular reactive oxygen species levels during foam cell development [23]. In that study, atherosclerosis development was characterized by macrophage autophagy inhibition and changes to the distribution and rate of macrophage polarization [23]. Therefore, we deduced that selective promotion of macrophage autophagy may reduce M1 macrophages, increase M2 macrophages, and alter macrophage foam cells to stabilize vulnerable atherosclerotic plaques.

Additionally, FTY720 can induce autophagy in some cancer cells [24–26]. However, little is known about whether FTY720 mediates macrophage autophagy and polarization in atherosclerosis. One previous study showed that FTY720 stimulated production of 27-hydroxycholesterol, an endogenous ligand of the liver X receptor, to cause liver X receptor-induced upregulation of ATP-binding cassette, subfamily A member 1 (ABCA1). It also conferred atheroprotective effects independent of sphingosine 1-phosphate receptor (S1PR) activation in human primary macrophages [27]. Furthermore, FTY720 inhibited inflammatory factors in endothelial and vascular smooth muscle cells through S1PR1 and S1PR3 and inhibited secretion of monocyte chemotactic protein 1 (MCP-1) by S1PR3 [14]. In vivo and in vitro experiments indicate that S1P mediates S1PR3 to recruit monocytes/macrophages and change smooth muscle cells to protect them from atherosclerosis [28]. Although FTY720 has a role in lipid metabolism and macrophage migration to reduce atherosclerosis, the drug may act as a functional antagonist or agonist, depending on S1P receptor subtype and cell or tissue target. Consequently, this study was designed to assess the roles of rapamycin and FTY720 on autophagy and polarization of macrophage cells and foam cells and to explore the S1PR macrophage foam cells to identify new approaches for reducing atherosclerosis.

## 2. Materials and Methods

### 2.1. Animal

B6D2F1 mice, aged 8 to 10 weeks, were purchased from Vital River (Beijing, China). Animals were treated according to the Guide for the Care and Use of Laboratory Animals. All animal experiments were performed under the Code of Practice, Harbin Medicine University Ethics Committees.

### 2.2. Peritoneal Macrophages and Culture

Mouse macrophages were isolated from the peritoneal cavity of B6D2F1 mice 3 days after treatment with 3% thioglycolate (T9032-500G, Sigma-Aldrich). Macrophages were cultured at 37°C in a 5% CO_2_ humidified incubator. Isolated macrophages were maintained in RPMI 1640 (22400089, Invitrogen) containing 10% fetal bovine serum (FBS, 04-001-1A, BI) and 50 *μ*g/mL penicillin/streptomycin (15140-148, Invitrogen) for 24 h. Next, the medium was changed, and the cells for RNA or protein analysis were removed 24 h after treatment with FTY720 (SML0700, Sigma-Aldrich) or rapamycin (ab120224, Abcam).

### 2.3. Immunofluorescence Staining

Cells were fixed in 4% paraformaldehyde for 20 min at 4°C. Next, cells were rinsed with 0.25% Triton X-100 in phosphate-buffered saline (PBS) and incubated with 70% alcohol for 5 min before being incubated with blocking buffer for 30 min at room temperature. For F4/80 staining, cells were incubated with F4/80 (565612, BD) overnight at 4°C and then counterstained with Hoechst 33342 (14533-100MG, Sigma-Aldrich). Lastly, cells were mounted on glass slides and examined using a Nikon microscope.

### 2.4. Western Blot

Cells were lysed using RIPA buffer (Sigma-Aldrich) containing Halt Protease and Phosphatase Inhibitor Cocktail (Thermo Fisher, Philadelphia, PA) for 20 min on ice. Samples were incubated at 70°C for 15 min in NuPAGE sample buffer (Life Technologies), and proteins were separated on NuPAGE 4% to 12% Bis-Tris gels before transfer to PVDF (Life Technologies) for immunoblotting. Several primary antibodies were used for detection: anti-LC3 (14600-1-AP, Proteintech), anti-TGF-*β* (ab66043, Abcam), anti-IL-6 (21865-1-AP, Proteintech), anti-COX2 (12375-1-AP, Proteintech), anti-Arg1 (16001-1-AP, Proteintech), and anti-glyceraldehyde-3-phosphate dehydrogenase (GAPDH, KC-5G4, KANGCHEN). All secondary antibodies used for visualization were either goat anti-mouse or goat anti-rabbit and were purchased from Abcam. Blots were developed with the SuperSignal West Pico Chemiluminescent Substrate or SuperSignal West Femto Maximum Sensitivity Substrate Kit (Thermo Fisher) and visualized by the ImageQuant LAS 4000 biomolecular imager (GE Healthcare Life Sciences, Pittsburgh, PA). Densitometry analysis was completed with the help of ImageJ software, which allows for quantification of band intensity. A rectangle was placed on each band, and the band intensity and background intensity were analyzed. Quantification was determined by subtracting band intensity from background intensity. Protein expression was corrected with a loading control such as GAPDH by dividing the protein densitometry value. All western blot data is presented as protein densitometry/control protein densitometry.

### 2.5. Foam Cell Formation

Copper ox-LDL (2 mg/mL) was purchased from Peking Union-Biology Co. Ltd (China). Mouse peritoneal macrophages were then seeded in a 12-well or 6-well cell culture (Corning) in RPMI 1640 media containing 10% fetal bovine serum (FBS) and allowed to adhere overnight. The next day, cells were treated with ox-LDL (150 *μ*g/mL) for 48 h.

### 2.6. Oil Red O Staining

Oil Red O stock solution (0.5%) in 100% isopropanol was diluted to 60% (vol/vol) isopropanol using distilled water. The solution was then filtered to remove particulates. After incubation with ox-LDL, cells were rinsed twice with PBS and fixed with 4% paraformaldehyde in PBS for 15 min at room temperature. Next, cells were rinsed twice with PBS and stained with a filtered Oil Red O solution for 1 h at room temperature. They were rinsed with distilled water and mounted using aqueous mounting media.

### 2.7. Real-Time Polymerase Chain Reaction

RNA samples from differentially treated macrophages were extracted using TRIzol Reagent (15596026, Invitrogen). cDNA was synthesized from mRNA using the High Capacity cDNA Reverse Transcription Kit (AT341-02, TransGen Biotech). Real-time PCR was performed using 1 *μ*L of cDNA, 10 *μ*L TransStart TM Top Green real time PCR SuperMix (AQ131, TransGen Biotech), and gene-specific primers in a 20 *μ*L reaction system on the CFX96 Real-Time System (Bio-Rad). Specific primers were obtained from a primer bank for mouse Arg1 (forward primer, 5′-CTCCAAGCCAAAGTCCTTAGAG; reverse primer, 5′-AGGAGCTGTCATTAGGGACATC), Mrc1 (forward primer, 5′-CTCTGTTCAGCTATTGGACGC; reverse primer, 5′-GGAATTTCTGGGATTCAGCTTC), iNOS (forward primer, 5′-GTTCTCAGCCCAACAATACAAGA; reverse primer, 5′-GTGGACGGGTCGATGTCAC), IL-6 (forward primer: 5′-CCAAGAGGTGAGTGCTTCCC; reverse primer, 5′-CTGTTGTTCAGACTCTCTCCCT), S1PR1 (forward primer, 5′-ATGGTGTCCACTAGCATCCC; reverse primer, 5′-CGATGTTCAACTTGCCTGTGTAG), S1PR2 (forward primer, 5′-ATGGGCGGCTTATACTCAGAG; reverse primer, 5′-GCGCAGCACAAGATGATGAT), S1PR3 (forward primer, 5′-TTTCATCGGCAACTTGGCTCT; reverse primer, 5′-GCTACGAACATACTGCCCTCC), S1PR4 (forward primer, 5′-GTCAGGGACTCGTACCTTCCA; reverse primer, 5′-GATGCAGCCATACACACGG), and S1PR5 (forward primer, GCTTTGGTTTGCGCGTGAG; reverse primer, 5′-GGCGTCCTAAGCAGTTCCAG). GAPDH was used as an internal control, and each sample was run in triplicate. The 2-ΔΔCt method was used to analyze qPCR gene expression data. Significance was assessed using two-tailed Student's* t*-test to compare the levels of DE genes for different groups (*P* < .05).

### 2.8. Statistical Analysis

Data were expressed as means ± standard deviations and were tested for normality by SPSS Statistics (*P* > .05). Then, ANOVA was performed on the data using SPSS Statistics. A value of* P* < .05 was considered to be statistically significant. A value of* P* < .01 was considered to be more statistically significant.

## 3. Results

### 3.1. Rapamycin Induces M1 Polarization by Increasing Macrophage Autophagy

**Figure 1:**
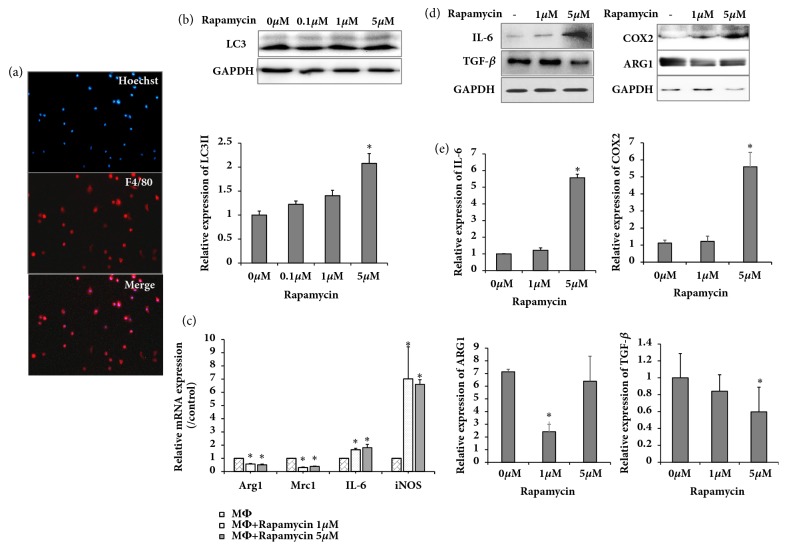
Rapamycin promoted autophagy in peritoneal macrophages and activated the M1 phenotype. (a) Peritoneal macrophages subjected to immunofluorescence staining by macrophage marker F4/80 antibody; (b) western blot showing LC3 expression in various rapamycin- (Rap-) treated groups, relative protein and GAPDH levels shown as histograms; (c) real-time PCR showing expression of Arg1, Mrc1, IL-6, and iNOS in various rapamycin-treated groups; (d) western blot showing expression of IL-6, TGF-*β*, COX2, and ARG1 in various rapamycin- (Rap-) treated groups; (e) relative protein and GAPDH levels shown as histograms; ^*∗*^*P* < .05 and ^*∗∗*^*P* < .01 vs. untreated controls. Results are representative of 3 independent experiments; each experiment was repeated 3 times.

Peritoneal macrophages were isolated as described previously. Immunofluorescence analysis showed a positivity rate of 87.6% for F4/80 (Figure 1(a)). To investigate the effect of autophagy on macrophages, expression of autophagic markers LC3I and LC3II in macrophages was analyzed. Cells were cultured with or without autophagy activator rapamycin for 24 h. After treatment, cells were harvested, and proteins were collected for western blot analysis. As shown in Figure 1(b), rapamycin increased expression of LC3-II at different doses. Furthermore, real-time PCR showed that IL-6 and iNOS (M1 markers) were increased whereas Arg1 and Mrc1 (M2 markers) were decreased (Figure 1(c)). Moreover, western blot analysis demonstrated that IL-6 and COX2 (M1 markers) were higher in rapamycin-treated compared with untreated macrophages. Meanwhile, expression of TGF-*β* and ARG1 (M2 markers) was lower in rapamycin-treated compared with untreated macrophages (Figures 1(d) and 1(e)). This finding suggested that autophagy could induce M1 polarization.

### 3.2. Rapamycin Does Not Affect Foam Cell Polarization

**Figure 2:**
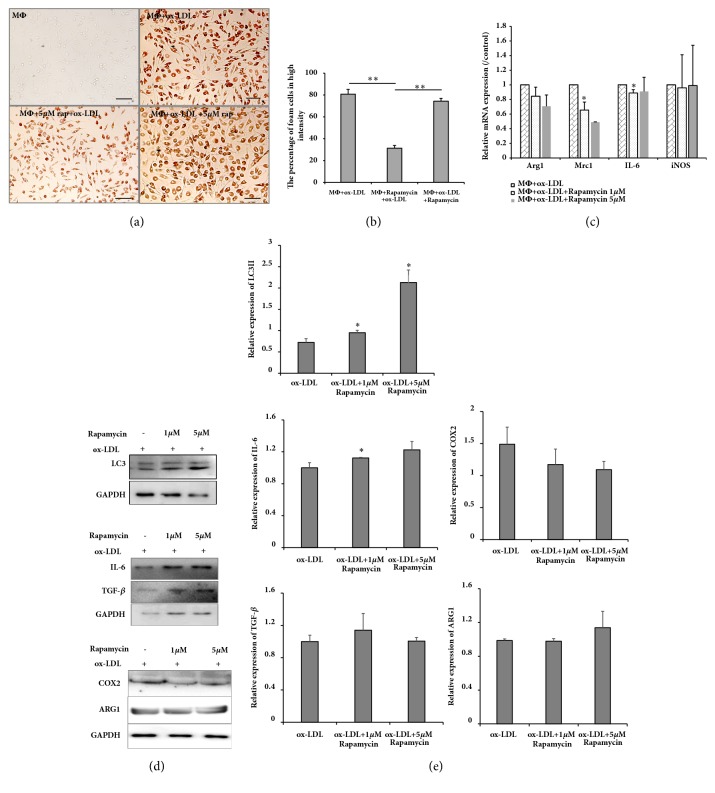
Rapamycin had no effect on foam cell polarization. (a) Oil Red O staining demonstrated that rapamycin inhibited macrophage transformation to foam cells but did not affect foam cells; (b) percentage of foam cells with high intensity from 3 experimental iterations: 6 views were analyzed from 2 randomly selected staining wells; ^*∗*^*P* < .05 and ^*∗∗*^*P* < .01 vs. ox-LDL; (c) real-time PCR showing Arg1, Mrc1, IL-6, and iNOS expression for various rapamycin-treated groups; (d) western blot showing expression of LC3, IL-6, COX2, TGF-*β*, and ARG1 in foam cells treated with various concentrations of rapamycin; (e) results shown representative of 3 independent experiments, each experiment repeated 3 times. Protein and GAPDH relative levels are shown as histograms.* P* < .05 and ^*∗∗*^*P* < .01 vs. untreated controls; MΦ, macrophage; bar, 100 *μ*m.

Macrophage foam cells indicate advanced stage atherosclerosis. To generate foam cells, macrophages were treated by ox-LDL for 48 h. Oil Red O staining showed that ox-LDL increased lipid accumulation and that this accumulation was decreased by rapamycin treatment (Figures 2(a) and 2(b)). To determine whether autophagy affected polarization of foam cells, rapamycin was used at various doses. First, western blot analysis demonstrated that autophagy marker LC3II was elevated in the presence of rapamycin. Next, real-time PCR showed that rapamycin had almost no effect on foam cell polarization (Figure 2(c)). Western blot analysis verified that TGF-*β*, ARG1 and COX2 expression were unchanged in rapamycin-treated foam cells; IL-6 increased slightly in foam cells treated with 1 *μ*M rapamycin (Figures 2(d) and 2(e)). However, lipid accumulation did not differ between rapamycin-treated foam cells and ox-LDL-treated foam cells (Figures 2(a) and 2(b)). Thus, autophagy appeared to have no effect on foam cell polarization.

### 3.3. FTY720 Reduces Lipid Accumulation by M2 Polarization of Foam Cells

**Figure 3:**
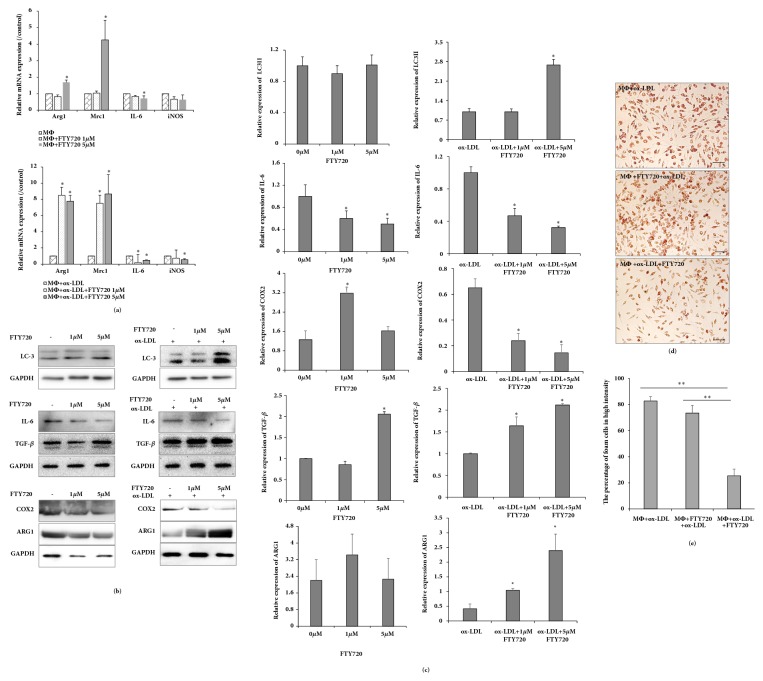
FTY720 reduces lipid accumulation by M2 polarization of foam cells. (a) Real-time PCR showing Arg1, Mrc1, IL-6, and iNOS expression with various concentrations of rapamycin treatment in macrophages and foam cells. (b) Western blot showing LC3, ARG1, COX2, IL-6, and TGF-*β* in macrophages and foam cells treated with various concentrations of rapamycin. (c) Results shown representative of 3 independent experiments, each experiment repeated 3 times. Data representative of protein and relative GAPDH are shown as histograms; ^*∗*^*P* < .05 vs. untreated controls. (d) Oil Red O staining demonstrating that FTY720 reduced lipid accumulation in macrophage foam cells; bar, 100 *μ*m. (e) Percentage of foam cells with high intensity from 3 experimental iterations: 6 views were analyzed from 2 randomly selected staining wells; ^*∗*^*P* < .05 and ^*∗∗*^*P* < .01 vs. ox-LDL.

A previous study reported that M2 polarization by S1P was responsible for the antiatherogenic properties of high-density lipoproteins in vivo [13]. FTY720 was not only an S1P analogue, but also an S1P receptor modulator that had contradicting roles in lipid metabolism and macrophage migration in atherosclerotic disease. To test the effect of FTY720 on polarization, FTY720-treated macrophages and foam cells were used. Real-time PCR showed that high-dose FTY720 treatment of macrophages increased Arg1 and Mrc1, reduced IL-6, and did not change iNOS. However, Arg1 and Mrc1 were increased, whereas IL-6 and iNOS were decreased in FTY720-treated foam cells (Figure 3(a)). Western blot demonstrated that TGF-*β* and ARG1 expression were increased in both FTY720-induced macrophages and FTY720-induced foam cells; however, ARG1 expression did not change in FTY720-treated foam cells (Figures 3(b) and 3(c)). Additionally, western blot was used to verify that IL-6 and COX2 were reduced in FTY720-treated macrophages and foam cells, but COX2 was not reduced in FTY720-induced macrophages. These results indicate that FTY720 could improve M2 polarization of macrophages and foam cells. Furthermore, LC3 expression was detected in macrophages and foam cells. However, LC3II expression differed in both cell types. LC3II expression was increased in FTY720-treated foam cells, but not in FTY720-treated macrophages (Figures 3(a) and 3(b)). Thus, FTY720 appeared to transform the polarization of foam cells. Additionally, lipid accumulation was decreased in FTY720-treated foam cells (Figures 3(d) and 3(e)), but not FTY720-treated macrophages. This finding suggests that FTY720 played a key role in M2 polarization to alleviate advances in atherosclerosis; autophagy may also promote M2 polarization.

### 3.4. S1PR Involved in FTY720 and Rapamycin Regulation of Foam Cells

**Figure 4:**
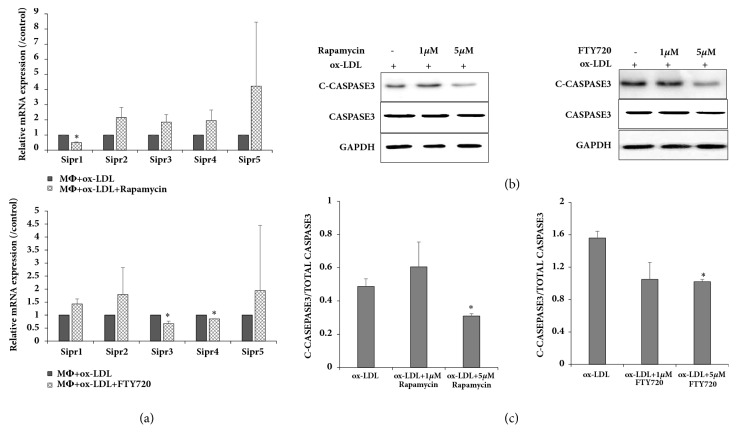
S1PR involvement in FTY720- and rapamycin-regulated foam cells; (a) real-time PCR analysis shown for S1pr1-5 expression in foam cells; (b) western blot showing apoptosis marker cleaved-CASPASE 3 and total CASPASE 3; (c) data representative of cleaved-CASPASE 3 levels relative to total CASPASE 3 levels shown as histograms. ^*∗*^*P* < .05 vs. untreated controls. Results shown are representative of 3 independent experiments; each experiment was repeated 3 times.

Previous research shows that S1P receptors are involved in atherosclerosis, possibly through endoplasmic reticulum (ER) stress, not PI3K/beclin1 or mTOR [29]. In this study, S1PR was detected in rapamycin-induced and FTY720-induced foam cells. Real-time PCR showed that S1pr1 expression was lower in rapamycin-treated compared with untreated foam cells (*P *< .05; Figure 4(a)). However, FTY720 inhibited expression of S1pr3 and S1pr4 in foam cells (Figure 4(a)).

Macrophage apoptosis in atherosclerotic plaques may increase plaque destabilization. Therefore, the next investigation aimed to determine whether FTY720-mediated M2 polarization caused macrophages to resist apoptosis. Expression of cleaved-CASPASE 3 was slightly decreased in 5 *μ*M FTY720-mediated foam cells but was unchanged in rapamycin-treated foam cells (Figures 4(b) and 4(c)). These results support the antiapoptotic and atheroprotective effects of FTY720.

## 4. Discussion

Atherosclerosis is characterized by persistent inflammation of the arterial wall. Macrophages, the most abundant immune cells in atherosclerotic plaques, can be transformed to foam cells by oxidized low-density lipoprotein (ox-LDL) induction, which accelerates atherosclerosis [1, 6–9]. Thus, macrophages and foam cells are crucial in different stages of atherosclerotic disease and, as such, are attractive targets for therapy. Moreover, macrophages are functionally complex in their adoption of multiple polarization states based on the composition of their microenvironment. Previous reports show that defective phagocytic clearance of macrophages promotes plaque necrosis and inhibition of autophagy by silencing ATG5 or other autophagy mediators to enhance protective processes in atherosclerosis [22]. Furthermore, mTOR mediates cross talk of macrophage polarization and autophagy in atherosclerosis [30]. However, the polarization and autophagy of foam cells in the pathogenesis of atherosclerosis remain largely unexplained. Understanding cross talk of autophagy and polarization regulation is crucial to understanding pathogenesis.

In our studies, we found that macrophage and foam cell autophagy produce different effects. Rapamycin is a recognized autophagy activator through its inhibition of mTOR signaling in various cells. By increasing LC3II expression, we first verified the increased autophagy induced by rapamycin in both macrophages and foam cells. Furthermore, our finding that rapamycin induced M1 polarization by increasing IL-6, iNOS and COX2 expression, while decreasing TGF-*β*, Arg1, and MRC1 expression in macrophages, is consistent with a previous report [31]. However, we found no effect on foam cell polarization. Moreover, Oil Red O staining showed decreased lipid accumulation in rapamycin-treated macrophages but not rapamycin-treated foam cells. This finding suggests that M1 macrophages may inhibit foam cell formation. M1 macrophages have been previously reported to promote inflammation through direct targeting of the NF-*κ*B pathway [32].

Atherosclerosis is a chronic immunoinflammatory disease [1]. Here, we found that M1 macrophages can enhance autophagy by inhibiting mTOR signaling, a key regulator of atherosclerosis. Thus, mTOR depletion stimulates M1 macrophages and suppresses early atherosclerosis. In this study, rapamycin also enhanced autophagy by inhibiting mTOR in foam cells, though it did not affect polarization or alter lipid load. In vivo results showed that rapamycin attenuated the macrophage death rate and delayed plaque progression through autophagy upregulation [23, 33, 34]. Wang and colleagues previously showed that cholesterol efflux was increased by autophagy in macrophage foam cells [35]. Hence, rapamycin-induced autophagy in foam cells delays intracellular lipid accumulation and may stabilize artery plaques through prevention of apoptosis and S1PR1 in advanced atherosclerosis.

FTY720, a novel S1P analogue [12] and a potent immunosuppressive drug, acted as a sphingosine kinase (SphK) 1 antagonist, growth inhibitor, and apoptosis inducer in various human cancer cell lines. In this study, FTY720 did not affect autophagy in macrophages, but its activity in suppressing proinflammatory factor IL-6 in macrophages was consistent with a previous study [36]. In animal models, FTY720 reduced atherosclerosis by suppressing monocyte/macrophage migration, inducing a regulatory T-cell response, and inhibiting effector T-cell responses [14, 15]. Moreover, the M1 phenotype marker-IL-6 was inhibited, whereas the M2 phenotype marker IL-4 was increased in peritoneal macrophage cells from FTY720-treated LDLR-deficient mice fed a cholesterol-rich diet [12]. In the present study, FTY720 also inhibited M1 phenotype markers IL-6, COX2, and iNOS, promoted M2 markers TGF-*β*, Arg1, and Mrc1 in macrophage foam cells, and reduced lipid accumulation in macrophage foam cells, as shown by Oil Red O staining. LC3-II was notably increased in FTY720-treated foam cells compared with FTY720-treated macrophages, which have been shown in some cancer studies to induce the autophagy pathway [24–26, 37–39]. Consequently, we deduced that FTY720 affected macrophage function and polarization through constriction of the M1 phenotype and activation of the M2 phenotype via the autophagy pathway to alleviate advanced atherosclerosis. Additionally, the finding that FTY20 inhibited S1PR3 and S1PR4 in macrophage foam cells was similar to previous reports that FTY20 could suppress S1PR1 and S1PR3 to decrease inflammatory factors and alleviate atherosclerosis [14, 28]. It is possible that FTY720 suppresses S1PR3 and S1PR4 to reduce lipid accumulation.

## 5. Conclusion

In summary, rapamycin induced autophagy, promoted the M1 phenotype, suppressed the M2 phenotype, and inhibited foam cell formation in peritoneal macrophages. Additionally, rapamycin activated autophagy in peritoneal macrophage foam cells but did not affect macrophage polarization or reduce lipid accumulation. Thus, the mTOR depletion of macrophage cells stimulated M1 macrophages and suppresses early atherosclerosis. FTY720 could promote the M2 phenotype and suppress the M1 phenotype in peritoneal macrophages and foam cells, but it only activated autophagy in peritoneal macrophage foam cells and alleviated lipid accumulation. FTY720 transformation of foam cells into the M2 phenotype occurred through the autophagy pathway and alleviated advanced atherosclerosis.

## Data Availability

The data used to support the findings of this study are included within the article.
